# Impact of acute *versus* prolonged exercise and dehydration on kidney function and injury

**DOI:** 10.14814/phy2.13734

**Published:** 2018-06-11

**Authors:** Coen C. W. G. Bongers, Mohammad Alsady, Tom Nijenhuis, Anouk D. M. Tulp, Thijs M. H. Eijsvogels, Peter M. T. Deen, Maria T. E. Hopman

**Affiliations:** ^1^ Department of Physiology Radboud Institute for Health Sciences Radboud University Medical Center Nijmegen The Netherlands; ^2^ Department of Physiology Radboud Institute for Molecular Life Sciences Radboud University Medical Center Nijmegen The Netherlands; ^3^ Department of Nephrology Radboud Institute for Molecular Life Sciences Radboud University Medical Center Nijmegen The Netherlands; ^4^ Research Institute for Sports and Exercise Sciences Liverpool John Moores University Liverpool United Kingdom

**Keywords:** Kidney function, kidney injury molecule‐1, neutrophil gelatinase‐associated lipocalin, renal damage, renal function

## Abstract

Exercise and dehydration may be associated with a compromised kidney function and potential signs of kidney injury. However, the kidney responses to exercise of different durations and hypohydration levels are not yet known. Therefore, we aimed to compare the effects of acute versus prolonged exercise and dehydration on estimated glomerular filtration rate (eGFR) and kidney injury biomarkers in healthy male adults. A total of 35 subjects (23 ± 3 years) were included and invited for two study visits. Visit 1 consisted of a maximal cycling test. On Visit 2, subjects performed a submaximal exercise test at 80% of maximal heart rate until 3% hypohydration. Blood and urine samples were taken at baseline, after 30 min of exercise (acute effects; low level of hypohydration) and after 150 min of exercise or when 3% hypohydration was achieved (prolonged effects, high level of hypohydration). Urinary outcome parameters were corrected for urinary cystatin C, creatinine, and osmolality. Subjects dehydrated on average 0.6 ± 0.3% and 2.9 ± 0.7% after acute and prolonged exercise, respectively (*P* < 0.001). The eGFR_cystatin C_ did not differ between baseline and acute exercise (118 ± 11 vs. 116 ± 12 mL/min/1.73 m^2^, *P* = 0.12), whereas eGFR_cystatin C_ was significantly lower after prolonged exercise (103 ± 16 mL/min/1.73 m^2^, *P* < 0.001). We found no difference in osmolality corrected uKIM1 concentrations after acute and prolonged exercise (*P* > 0.05), and elevated osmolality corrected uNGAL concentrations after acute and prolonged exercise (all *P*‐values < 0.05). In conclusion, acute exercise did barely impact on eGFR_cystatin C_ and kidney injury biomarkers, whereas prolonged exercise is associated with a decline in eGFR_cystatin C_ and increased biomarkers for kidney injury.

## Introduction

Strenuous exercise increases the perfusion of active muscles, whereas the perfusion of body organs such as the kidneys may decrease up to 25% of resting levels (Poortmans 1984; McAllister 1998). Furthermore, exercise increases the metabolic heat production, resulting in an elevated sweat rate and concomitant dehydration leading to a lower extracellular volume (Gonzalez‐Alonso 2012). It has been hypothesized that the decreased renal blood flow and lower circulatory blood volume may attenuate kidney function and induce ischemic kidney stress or even “temporary” kidney injury (Bonventre 1988; Lippi et al. 2008). Furthermore, literature suggested that exercise‐induced dehydration, heat stress, inflammation, and oxidative stress might also influence kidney function and induce kidney stress (Otani et al. 2013; Hewing et al. 2015).

Prolonged exercise accompanied with dehydration stimulates the secretion of arginine vasopressin (AVP) (Boone and Deen 2008; Knepper et al. 2015) and activates the renin–angiotensin–aldosteron system (RAAS) (Atlas 2007), both stimulate the renal reabsorption of water and sodium chloride. The increased energy‐demanding renal sodium uptake and the reduced renal perfusion with excessive dehydration may induce ischemic kidney injury (Roncal‐Jimenez et al. 2015). Increased urinary levels of kidney damage biomarkers (kidney injury molecule 1 (KIM1) and neutrophil gelatinase‐associated lipocalin (NGAL)) were found after completing a (ultra)marathon (McCullough et al. 2011; Lippi et al. 2012) and after prolonged walking exercise (Bongers et al. 2017). However, the interpretation of these studies is difficult, as urinary KIM1 and NGAL concentrations were not corrected for elevated urine density as an effect of dehydration. Some studies corrected for changes in urinary density using urinary creatinine concentration (Junglee et al. 2012; Lippi et al. 2012), which may be problematic because of exercise‐induced muscle breakdown (Junglee et al. 2012). As a result, it is hard to distinguish whether observed changes are the effect of exercise or due to an increased urine concentration because of dehydration. In addition, previous studies primarily focused on the effects of prolonged exercise on kidney function and injury, but the acute effects of a short bout of exercise, with less dehydration, remain unknown.

Therefore, the aim of this study was to assess and compare the effects of acute versus prolonged exercise on eGFR and kidney injury biomarkers in healthy male adults in well‐controlled laboratory circumstances. To account for exercise‐induced changes in urinary concentration, uNGAL and uKIM1 will be corrected for cystatin C, creatinine, and osmolality. We hypothesized that acute exercise will not impact on eGFR and kidney injury, whereas prolonged exercise will result in a decreased eGFR and the presence of biomarkers for kidney injury. Furthermore, we hypothesize that higher levels of hypohydration will augment the effects on eGFR and kidney injury biomarkers.

## Methods

### Subjects

A total of 35 male subjects (23 ± 3 years, 22.3 ± 3.6 kg/m^2^) between 18 and 30 years were included in this study. Subjects with a history of kidney disease or a baseline eGFR <90 mL/min were excluded for participation. Furthermore, patients with thyroid disease and autoimmune disorders were excluded for participation. The study was approved by the Medical Ethical Committee of the Radboud university medical center (CMO: 2015‐1649), and all participants gave written informed consent prior to participation. Furthermore, the study was conducted under the provisions of the Declaration of Helsinki.

### Study design

All subjects were invited for two study visits, separated by at least 5 days, to prevent interference of the two visits. After given informed consent, subjects were medically screened to control for exclusion criteria and a venous blood sample was taken. Thereafter, a maximal exercise test was performed to determine subject's physical fitness level (VO_2_ max) and maximal heart rate (HR max). The second study visit consisted of a submaximal exercise test on a cycle ergometer for 150 min or until 3% hypohydration. At baseline, after 30 min of exercise (effects of acute exercise) and directly after exercise (effects of prolonged exercise), a blood and urine sample were taken and dry‐toweled nude body mass was measured. Subjects were instructed to refrain from alcohol and caffeine consumption and heavy physical exercise 48 h prior to the experiment. Furthermore, subjects were instructed to register all fluid intake 24 h prior to the second study visit. To ensure that subjects were well hydrated before the exercise test, subjects were asked to drink 0.5 L of water 2 h prior to the test (Casa et al. 2000).

### Study protocol

#### Study visit 1 – medical screening + maximal exercise test

The medical screening consisted of a medical history check, a 12‐lead ECG and physical examination. Subsequently, a venous blood sample was taken to determine the serum creatinine concentration, which was used to assess kidney function in rest by calculating the eGFR_creatinine_. Furthermore, subjects were asked to complete the Short QUestionnaire to ASsess Health enhancing physical activity (SQUASH) (Wendel‐Vos et al. 2003). This questionnaire takes into account sport activities as well as other daily activities such as work, housekeeping and leisure time activities. Subjects completed the questionnaire concerning an average week of the past 6 months.

Furthermore, subjects completed a stepwise incremental cycling exercise protocol, in which the workload increased with 25 W per minute until volitional exhaustion, to determine subject's physical fitness (VO_2_ max), HR max, and maximal workload. Directly after volitional exhaustion we determined the rate of perceived exertion using a 10‐point category Borg scale (Borg 1982) and the blood lactate level using a fingertip capillary measurement (Lactate Pro, ARKRAY Europe, Amstelveen, The Netherlands).

#### Study visit 2 – submaximal exercise test

During the second study visit, subjects performed a submaximal exercise test on a cycle ergometer at an exercise intensity of 80% of the HR max. At baseline the nude body mass, equipped body mass (body mass including sportswear and HR monitor), blood pressure, and resting HR were measured. Furthermore, a venous blood sample was taken and subjects were asked to provide a urine sample and subsequently empty their bladder. Thereafter, subjects started the submaximal exercise test, which consisted of two parts. In the first part, subjects exercised at 80% of HR max for 30 min at a frequency of 60–80 repetitions per minute in an ambient temperature of 20°C. In the first 5 min of the exercise test, the workload was increased until the subjects reached 80% of their maximal heart rate. Thereafter, the workload was adjusted to maintain a constant exercise intensity of 80%. After 30 min, the baseline measurements were repeated and blood and urine samples were taken to assess the acute effects of exercise. Subsequently, subjects got dressed with a thermo suit (Craft active Basic, Craft, Beverly, Massachusetts, USA) and started the second part of the exercise protocol, in which the ambient temperature was elevated to 25°C. Subjects started cycling at 80% of HR max and continued exercise for another 120 min or until 3% hypohydration (defined as a body mass loss of 3%) was achieved. The rate of perceived exertion (RPE) was measured on a 6–20 category Borg scale. Furthermore, the equipped body mass, ambient temperature, and relative humidity were measured every 15 min. Directly after completing the submaximal exercise test, the blood pressure was measured and blood and urine samples were taken again. Thereafter, the nude body mass was measured to determine the relative body mass loss (hypohydration).

### Study parameters

#### Fluid balance

Nude body mass was measured at baseline, after 30 min and directly after finishing the submaximal exercise test. Subsequently, the exact level of hypohydration was calculated as the relative change in nude body mass (in %). Furthermore, all subjects received written and individual oral instruction about the registration of their fluid intake in a diary. Subjects were allowed to drink ad libitum, as long as they registered the time (in blocks of 1 h), amount (standardized sized cups, bottles, etc.), and type (water, sports drink, tea) of their individual fluid intake 24 h preceding the submaximal exercise test.

#### Blood sample

A blood sample was taken at baseline, after 30 min and directly after completing the submaximal exercise test. Serum creatinine and cystatin C concentrations were determined to examine kidney function. In literature, the Chronic Kidney Disease Epidemiology Collaboration formula (CKD‐EPI) based on creatinine is often used to determine kidney function (Levey et al. 2009). However, serum creatinine levels increase during exercise due to exercise‐induced muscle breakdown. In contrast, cystatin C is independent of muscle mass, age and gender, and serum levels are not influenced by exercise (Inker et al. 2012). Therefore, we used the CKD‐EPI formula based on creatinine (Levey et al. 2009) as well as the CKD‐EPI formula based on cystatin C (Inker et al. 2012) to calculate the eGFR at rest and after acute and prolonged exercise. Additionally, serum osmolality and plasma sodium concentration were measured as indices for dehydration. Plasma hematocrit and hemoglobin levels were measured and used to calculated the relative changes in plasma volume (in %) based on the Dill and Costil formula (Dill and Costill 1974). Furthermore, the plasma renin activity (PRA) and copeptin concentration were measured, to assess the hormonal responses to changes in the volume and osmolality balances, respectively. Arginine vasopressin (AVP) is relatively difficult to measure because of its short half‐life and its interaction with blood platelets (Morgenthaler et al. 2006). Copeptin and AVP are derived from the same preprohormone and thus synthesized and secreted in equal molar amounts. Copeptin, however, has a longer half‐life and can be measured more easily than AVP (Morgenthaler et al. 2006). Therefore, we measured copeptin as a surrogate marker for AVP secretion (Morgenthaler 2010).

#### Urine sample

At baseline, after 30 min and directly after completing the submaximal exercise test, all subjects provided a urine sample to examine fluid balance and kidney responses. Urinary cystatin C concentration was measured using the nephelometric method (Behring Nephelometer II, Siemens Healthcare, Den Haag, The Netherlands). The urinary creatinine concentration was measured using an enzymatic assay (Cobas C6000, Roche Diagnostics, Indianapolis, USA). Urine osmolality was examined using an osmometer (Advanced Model 3320 Micro‐Osmometer, Osmometer, Advanced Instruments, Norwood, USA).

In order to determine kidney injury in response to exercise, we measured urinary concentrations of KIM1 and NGAL (both monomeric and dimeric) in duplicate using the previously described sandwich ELISA assay (E‐EL‐H0186 and E‐EL‐H0096, Elabscience Biotechnology, Wuhan, China) (Han et al. 2002; van Timmeren et al. 2007). Furthermore, uKIM1 and uNGAL concentrations were corrected for urinary cystatin C, creatinine, and osmolality. The corrected uKIM1 and uNGAL data were calculated by dividing the individual uncorrected data by the corresponding cystatin C levels, creatinine levels, and the urine osmolality. Additionally, urinary albumin (uAlbumin) and glucose (uGlucose) concentrations were measured, and corrected as well, to examine the effects of acute exercise and dehydration on acute kidney injury.

#### Exercise intensity

Subjects HR was measured continuously every 15 sec throughout the test using a two‐channel chest band system (Polar RS400, Polar, Oy, Kempele, Finland). Subsequently, the exercise intensity was calculated by expressing the HR as a percentage of HR max.

### Statistical analysis

The statistical analyses were conducted using the Statistical Package for Social Sciences (SPSS version 20, Armonk, NY, USA), in which the level of significance was set at *P* < 0.05. Data were checked for normality using the Shapiro–Wilk test. In case of a non‐Gaussian distribution, the statistical analysis was performed using the nonparametric equivalents. Normally distributed data were presented as mean ±  standard deviation (SD), whereas non‐Gaussian distributed date were presented as median (interquartile range). A repeated measures ANOVA was used to assess differences in fluid balance, kidney function, and kidney injury over time, in which a post hoc Bonferroni correction was used to determine individual differences between baseline, acute exercise, and prolonged exercise. Non‐Gaussian distributed data were tested using a Friedman test, followed by a Wilcoxon signed‐rank test. Furthermore, we have calculated the absolute change in uKIM1 and uNGAL compared to baseline for both acute (∆ acute exercise) and prolonged exercise (∆ prolonged exercise). Subsequently, we used a paired *t*‐test or Wilcoxon signed‐rank test to assess whether there is a difference between ∆ acute exercise and ∆ prolonged exercise. A Pearson or Spearman (for non‐Gaussian distributed data) correlation coefficient was used to assess the relation between kidney function and injury and level of dehydration.

## Results

### Subjects & exercise characteristics

An overview of subject characteristics is shown in Table 1. Because of a vasovagal syncope in response to the venous blood sample, one subject did not perform the submaximal exercise test. Average fluid intake 24 h prior to the test was 2956 ± 947 mL. The average exercise duration during the submaximal exercise was 107 ± 16 min, in which subjects cycled at an exercise intensity of 79.3 ± 1.4% of HRmax. The average workload in the first part was significantly higher compared to the second part of the submaximal exercise test (152 ± 30 W vs. 116 ± 36 W, *P* < 0.001), with an average exercise intensity of 79.8 ± 3.5% and 81.1 ± 1.0% for the first and second part, respectively (*P* = 0.016). Furthermore, the ambient temperature and relative humidity were, respectively, 20 ± 1°C and 61 ± 14% for the first part and 25 ± 1°C and 59 ± 12% for the second part of the submaximal exercise test.

**Table 1:** Subject characteristics and results of maximal exercise test (*n* = 34)

Parameter	Total group (*n* = 34)
Subject characteristics	
Age (years)	22.8 ± 2.9
Length (m)	1.83 ± 0.06
Body mass (kg)	74.6 ± 10.5
BMI (kg/m^2^)	22.5 ± 3.6
Systolic blood pressure (mmHg)	128 ± 10
Diastolic blood pressure (mmHg)	69 ± 9
Resting heart rate (bpm)	66 ± 14
Serum Creatinine (μmol/L)	84.3 ± 8.9
eGFR_creatinine_ (mL/min/1.73 m^2^)	110.8 ± 11.0
Activity score (au)	8579 ± 4127
Maximal exercise test	
VO_2_ max (mL/min/kg)	56.6 ± 10.6
HR max (bpm)	194 ± 9
Maximal workload (W)	338 ± 55
Blood lactate level (mmol/L)	12.9 ± 1.8
RER (ratio: VCO_2_/VO_2_)	1.18 ± 0.07
Rate of perceived exertion (au)	8.2 ± 1.2

Subject characteristics for the total group. Data were presented as mean ± SD. MET, Metabolic equivalent of task, eGFR, estimated glomerular filtration ratio, bpm, beats per minute, au, arbitrary unit.

### Fluid balance

Fluid balance data are shown in Table 2. Relative body mass loss after acute and prolonged exercise was 0.6 ± 0.3% and 2.9 ± 0.7%, respectively (*P* < 0.001), while the decrease in plasma volume was also higher after prolonged exercise (*P* < 0.001). An exercise‐induced increase in urine osmolality was observed after both acute and prolonged exercise (both *P*‐values <0.001). No differences in serum sodium concentration and serum osmolality were found after acute exercise (*P* > 0.05), whereas sodium concentration and serum osmolality increased after prolonged exercise (*P* < 0.001). Plasma copeptin concentration after acute exercise did not increase (*P* = 0.07), whereas a significant increase was found after prolonged exercise (*P* < 0.001). Furthermore, baseline PRA levels increased after both acute and prolonged exercise (both *P*‐values <0.001), with higher PRA levels after prolonged exercise compared to acute exercise (*P* < 0.001).

**Table 2:** Fluid balance responses

Parameter	Baseline	Acute exercise	Prolonged exercise	*P*‐value
Relative body mass loss (%)	–	0.6 ± 0.3^a^	2.9 ± 0.7^a^^,^ b	**<0.001**
Plasma hemoglobin (mmol/L)	9.2 ± 0.7	9.6 ± 0.6^a^	9.7 ± 0.6^a^^,^ b	**<0.001**
Plasma hematocrit (L/L)	0.47 ± 0.03	0.48 ± 0.03^a^	0.49 ± 0.03^a^	**<0.001**
Plasma volume loss (%)	–	3.5 ± 2.1	4.8 ± 2.2^b^	**<0.001**
Urine Osmolality (mOsm/kg)	364 (201–624)	585 (360–735)^a^	837 (728–961)^a^^,^ b	**<0.001**
Serum Osmolality	293 ± 7	294 ± 6	300 ± 6^a^^,^ b	**<0.001**
Serum Sodium	142.2 ± 2.3	141.9 ± 2.7	144.0 ± 2.6^a^^,^ b	**<0.001**
Plasma copeptin (pmol/L)	4.6 (3.3–7.4)	6.6 (4.2–13.5)	35.9 (25.7–47.6)^a^^,^ b	**<0.001**
Plasma renin activity (pmol/L)	1.6 (1.1–2.6)	4.5 (3.2–5.7)^a^	13.4 (9.9–19.0)^a^^,^ b	**<0.001**

Bold indicates significant difference values.

Data were presented as mean ± SD or median (interquartile range).

a Significantly different from baseline.

b Different from acute exercise.

### Kidney function

**Figure 1:**
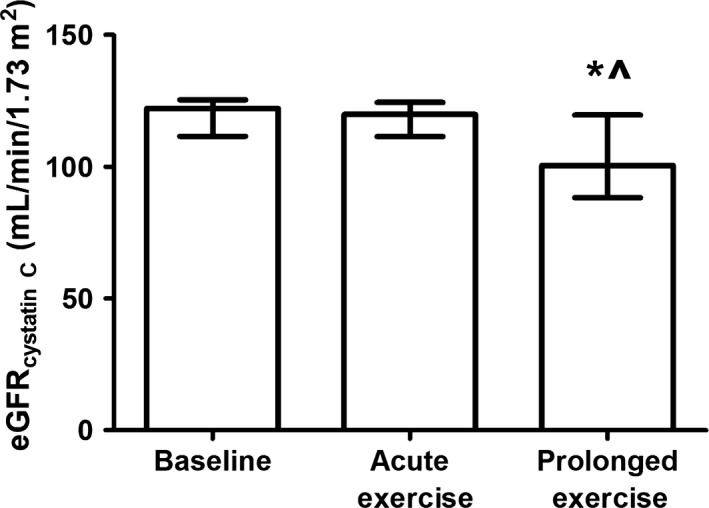
The estimated glomerular filtration ratio calculated with the cystatin C formula at baseline and after acute and prolonged exercise. A Friedman test was used to examine differences over time, whereas a Wilcoxon signed‐rank test was used to assess differences acute and prolonged exercise. Data were presented as median with interquartile range. *Represents a difference between acute and prolonged exercise.

An exercise‐induced increase in serum creatinine concentration was found after both acute (*P* = 0.011) and prolonged exercise (*P* < 0.001), with higher levels after prolonged compared to acute exercise (*P* < 0.001). Serum cystatin C levels were comparable between baseline and acute exercise (*P* = 0.36), whereas an increased cystatin C concentration was found after prolonged exercise (*P* < 0.001). Baseline eGFR_creatinine_ was 114 (102–123) mL/min/1.73 m^2^ and decreased to 109 (95–122) mL/min/1.73 m^2^ after acute exercise (*P* = 0.009), with a further decrease to 98 (82–105) mL/min/1.73 m^2^ after prolonged exercise (*P* < 0.001). In contrast, baseline eGFR_cystatin C_ did not change after acute exercise (118 ± 11 mL/min/1.73 m^2^ vs. 116 ± 12 mL/min/1.73 m^2^, *P* = 0.12), whereas a significant decrease was found after prolonged exercise (103 ± 16 mL/min/1.73 m^2^, *P* < 0.001, Fig. 1). Furthermore, no correlation was found between eGFR_cystatin C_ and level of hypohydration after acute (*R* ^2^ = 0.03, *P* = 0.30) and prolonged exercise (*R* ^2^ = 0.01, *P* = 0.54).

### Urinary kidney injury markers

Due to technical difficulties the data analysis of one of the badges of uKIM1 failed and uKIM1 cannot be determined in *n* = 9 subjects. Furthermore, *n* = 3 subjects were not able to provide a urine sample after 30 min of exercise. A significant increase in urinary creatinine and cystatin C concentration was found after acute and prolonged exercise (all *P*‐values <0.05), with higher levels after prolonged compared to acute exercise (Table 3).

**Table 3:** Urinary outcome parameters

Parameter	Baseline	Acute exercise	Prolonged exercise	*P*‐value
uCystatin C (mg/L)	0.01 (0.01–0.04)	0.03 (0.01–0.08)^a^	0.15 (0.09–0.26)^a^^,^ b	**<0.001**
uCreatinine (mmol/L)	5.0 (3.3–14.5)	9.2 (5.5–19.1)^a^	26.3 (20.5–37.8)^a^^,^ b	**<0.001**
uAlbumin (mg/mL)	3.9 (2.1–7.1)	10.0 (3.8–23.2)^a^	32.5 (16.4–50.1)^a^^,^ b	**<0.001**
uAlbumin (mg/μg Cystatin C)	308 (130–425)	313 (150–821)	161 (119–553)	0.14
uAlbumin (mg/mg Creatinine)	6.0 (3.9–11.0)	7.3 (5.3–14.4)	7.9 (6.0–12.8)^a^	**<0.001**
uAlbumin (μg/mOsm)	11.7 (7.6–21.0)	17.5 (9.6–36.4)^a^	35.1 (20.9–63.4)^a^^,^ b	**<0.001**
uGlucose (mmol/L)	0.11 (0.10–0.28)	0.17 (0.11–0.33)^a^	0.50 (0.33–0.79)^a^^,^ b	**<0.001**
uGlucose (mmol/mg Cystatin C)	5.7 (2.7–12.0)	5.5 (3.5–17.0)	3.1 (2.1–4.0)^a^^,^ b	**<0.001**
uGlucose (mmol/g Creatinine)	0.19 (0.15–0.24)	0.18 (0.17–0.22)	0.16 (0.13–0.18)^a^^,^ b	**0.023**
uGlucose (μmol/mOsm)	3.4 (2.4–4.8)	3.4 (2.7–5.1)	5.4 (4.4–8.9)^a^^,^ b	**0.026**

Bold indicates significant difference values.

Data were presented as mean ± SD or median (interquartile range).

a Significantly different from baseline.

b Different from acute exercise.

### Urinary NGAL

**Figure 2:**
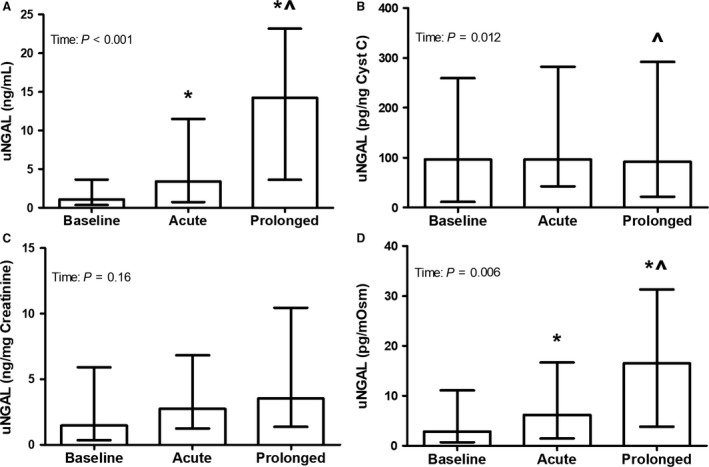
Urinary NGAL concentration uncorrected (A), as well as after correction for cystatin C (B), creatinine (C), and osmolality (D), at baseline and after acute and prolonged exercise (*n* = 31). A Friedman test was used to examine differences in uNGAL over time, whereas a Wilcoxon signed‐rank test was used to assess differences acute and prolonged exercise. Data were presented as median (interquartile range) for uncorrected and creatinine and osmolality corrected. *Represents a significant difference from baseline, and ^ represents a difference from acute exercise.

The uncorrected uNGAL concentration increased after both acute (*P* = 0.001) and prolonged exercise (*P* < 0.001), with higher levels after prolonged exercise (*P* = 0.001, Fig. 2).The cystatin C corrected uNGAL concentration did not differ after acute or prolonged exercise (both *P*‐values >0.05), with lower levels after prolonged compared to acute exercise (*P* = 0.044). No difference in creatinine corrected uNGAL (*P* = 0.16) concentrations were found across measurements. The osmolality corrected uNGAL concentration also increased after both acute (*P* = 0.018) and prolonged exercise (*P* < 0.001), with higher levels after prolonged exercise (*P* = 0.022). The ∆ prolonged exercise was significantly higher compared to ∆ acute exercise for uncorrected uNGAL and cystatin C and osmolality corrected uNGAL (all *P*‐values <0.05), whereas no difference between acute and prolonged exercise in change in creatinine corrected uNGAL was found (*P* = 0.58, Table 4). After both acute and prolonged exercise, no correlation was found between the uncorrected and corrected uNGAL concentrations and hypohydration level and absolute body mass loss (all *P*‐values>0.05).

**Table 4:** Changes in uKIM1 and uNGAL compared to baseline

Parameter	∆ Acute exercise	∆ Prolonged exercise	*P*‐value
uKIM1 (ng/mL)	0.7 (−0.2–2.1)	3.0 (0.8–7.6)	**0.003**
uKIM1 (pg/ng Cystatin C)	4.2 ± 72.0	−20.1 ± 59.2	**0.01**
uKIM1 (ng/mg Creatinine)	0.1 (−0.9–1.0)	0.1 (−0.9–1.5)	0.69
uKIM1 (pg/mOsm)	0.8 (−1.1–2.4)	2.7 (−0.1–8.2)	**0.022**
uNGAL (ng/mL)	1.0 (0.0–8.0)	11.1 (0.7–22.6)	**0.001**
uNGAL (pg/ng Cystatin C)	30.1 (−49.5–84.3)	−15.1 (−86.5–55.8)	**0.044**
uNGAL (ng/mg Creatinine)	1.8 ± 4.6	2.9 ± 8.8	0.58
uNGAL (pg/mOsm)	2.7 (−0.5–8.01)	8.0 (0.2–28.6)	**0.022**

Bold indicates significant difference values.

Data were presented as mean ± SD or median (interquartile range).

### Urinary KIM1

**Figure 3:**
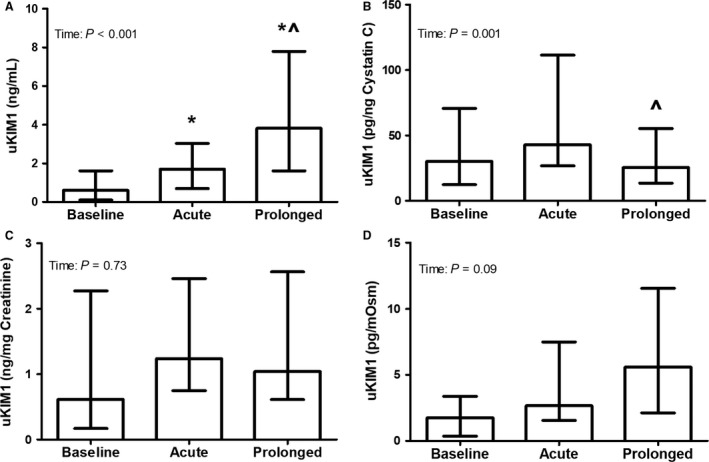
Urinary KIM1 concentration uncorrected (A), as well as after correction for cystatin C (B), creatinine (C), and osmolality (D), at baseline and after acute and prolonged exercise (*n* = 22). A Friedman test was used to examine differences in uKIM1 over time, whereas a Wilcoxon signed‐rank test was used to assess differences acute and prolonged exercise. Data were presented as median (interquartile range). *Represents a significant difference from baseline, and ^^^represents a difference from acute exercise.

The uncorrected uKIM1 concentration increased after both acute (*P* = 0.021) and prolonged exercise (*P* < 0.001), with higher levels after prolonged exercise (*P* = 0.003, Fig. 3). Cystatin C corrected uKIM1 was comparable after acute exercise (*P* = 0.52) and prolonged exercise (*P* = 0.062), with lower levels after prolonged compared to acute exercise (*P* = 0.003). Creatinine and osmolality corrected uKIM1 levels did not differ across measurements (*P* = 0.73 and *P* = 0.09, respectively). The ∆ prolonged exercise was significantly higher compared to ∆ acute exercise for uncorrected uKIM1 and cystatin C and osmolality corrected uKIM1 (all *P*‐values <0.05), whereas no difference between acute and prolonged exercise in absolute change in creatinine corrected uKIM1 was found (*P* = 0.69, Table 4). A weak, but statistically significant, negative correlation was found between the uncorrected uKIM1 concentration and level of hypohydration after acute exercise (*R* ^2^ = −0.46, *P* = 0.029) and a positive correlation was found between creatinine corrected uKIM1 and level of hypohydration after prolonged exercise (*R* ^2^ = 0.45, *P* = 0.022).

### Urinary albumin

The uncorrected uAlbumin concentration significantly increased after both acute (*P* < 0.001) and prolonged exercise (*P* < 0.001), with higher levels after prolonged exercise compared to acute exercise (*P* < 0.001). Cystatin C corrected uAlbumin levels did not differ across measurements (*P* = 0.14). Creatinine corrected uAlbumin levels did not differ after acute exercise (*P* = 0.13), whereas increased levels were found after prolonged exercise (*P* = 0.005). The osmolality corrected uAlbumin concentration was increased after both acute (*P* = 0.028) and prolonged exercise (*P* < 0.001), with higher levels after prolonged exercise (*P* = 0.027).

### Urinary glucose

The uncorrected uGlucose concentration increased after both acute (*P* = 0.024) and prolonged exercise (*P* < 0.001), with higher levels after prolonged exercise (*P* < 0.001). Furthermore, the cystatin C, creatinine, and osmolality corrected uGlucose concentration did not differ from baseline after acute exercise (all *P*‐values >0.05), whereas the cystatin C, creatinine, and osmolality corrected uGlucose levels were higher compared to baseline after prolonged exercise (all *P*‐values <0.05).

## Discussion

This is the first study that makes a direct comparison of the effects of acute versus prolonged exercise on eGFR and kidney injury biomarkers in healthy male adults. Furthermore, we are the first to use urinary cystatin C and osmolality to correct urinary biomarkers for changes in hydration status. We found that the eGFR_cystatin C_ did not change after acute exercise, whereas it significantly decreased after prolonged exercise. Furthermore, the uncorrected uKIM1 concentration and uncorrected and osmolality corrected uNGAL concentrations were elevated after both acute and prolonged exercise, with higher levels after prolonged compared to acute exercise. Moreover, ∆ prolonged exercise was significantly higher compared to ∆ acute exercise for uncorrected, cystatin C corrected and osmolality corrected uKIM1 and uNGAL. These results suggest that acute exercise as well as prolonged exercise may be associated with kidney injury, in which lower levels of kidney injury biomarkers were found after acute compared to prolonged exercise.

The absence of a decrease in eGFR_cystatin C_ after a short bout of exercise suggests that the kidneys are well able to maintain kidney function in response to exercise and small perturbations in fluid balance. Literature reveals that the filtration fraction increases during exercise in response to a drop in renal blood flow, in which the increase in filtration fraction is caused by an increased vasoconstriction of the efferent arteriole (Poortmans 1984). As a result, the secretion of nitric oxide and prostaglandin E2 by the macula densa is upregulated, which results in a dilation of both the afferent and efferent arteriole and restoration of GFR (Poortmans 1984; Poortmans and Vanderstraeten 1994; Breyer and Breyer 2000). After prolonged exercise we did find a decrease in eGFR_cystatin C_, which is in line with previous studies with long distance runners and cyclists that demonstrated similar decreases in eGFR_creatinine_ postexercise, which were restored 24 h postexercise (Neumayr et al. 2005; Tian et al. 2011; Hewing et al. 2015). It has been suggested that the transient alterations in eGFR are associated with the postexercise hydration status, but it may also be affected by exercise‐induced inflammation or oxidative stress (Hewing et al. 2015). We did not find a correlation between the level of hypohydration and eGFR_cystatin C_ after both acute and prolonged exercise. The strain of dehydration, inflammation, and oxidative stress is lower after acute compared to prolonged exercise, which might suggest that the kidneys are well able to preserve eGFR_cystatin C_ after acute exercise, whereas eGFR_cystatin C_ declines after prolonged exercise.

The measurement of urinary biomarkers is likely to be influenced by changes in hydration status. Dehydration may impact urine concentration, which subsequently may overestimate the concentration of injury markers. Therefore, it is necessary to correct urinary biomarkers for changes in hydration status. We used urinary creatinine, cystatin C, and osmolality to correct our findings. Previous studies demonstrated, however, that serum and urine concentrations of creatinine may increase as a consequence of exercise‐induced muscle breakdown (Junglee et al. 2012). On the other hand, urinary cystatin C levels may slightly increase as a consequence of a decreased proximal reabsorption due to kidney stress (Conti et al. 2006). We used urinary cystatin C and creatinine concentrations to correct for changes in hydration status, while these correction methods both have limitations, since it may lead to an underestimation of the true effect of exercise on kidney injury. Alternatively, urine osmolality may be a better option to correct the data for hydration status. The urine osmolality is the most accurate measurement of total solute concentration and it therefore provides the best measurement of the kidney's concentrating ability (Armstrong 2005). As a result, the urine osmolality has previously been established as a valid measure for hydration status (Kavouras 2002), which might be used as a correction method. Therefore, we will discuss our results with respect to kidney injury based on the osmolality corrected data.

We found increased urine osmolality corrected uNGAL levels after both acute and prolonged exercise, whereas the urine osmolality corrected uKIM1 concentration tended to be higher after prolonged exercise. These results suggest an exercise‐induced development of kidney injury as a consequence of exercise‐induced kidney stress. This is further supported by increased osmolality corrected uAlbumin and uGlucose levels after acute and prolonged exercise, which suggests that the proximal reabsorption of both substances is deteriorated as a consequence of kidney stress (Brodehl et al. 1987; Nauta et al. 2013). In resting conditions, the proximal tubules almost completely reabsorb the NGAL that is produced continuously at low levels by neutrophils of different tissues (i.e., colon, trachea, and kidney epithelium) (Schmidt‐Ott 2011; Helanova et al. 2014). Additionally, the distal tubules secrete low levels of NGAL as well, resulting in low urinary concentrations (Schmidt‐Ott 2011; Martensson and Bellomo 2014). In case of kidney stress, the proximal tubular uptake of NGAL is impaired and the NGAL expression and release are upregulated in the distal tubule (Devarajan 2008; Schmidt‐Ott 2011). Both will increase the urinary excretion of NGAL, but the upregulated secretion of NGAL by the distal tubules is the primary source (Helanova et al. 2014). Therefore, the elevated osmolality corrected uNGAL levels after acute and prolonged exercise, as found in our study, suggest proximal tubular injury. Our findings are in line with previous studies that demonstrated increased uKIM1 and uNGAL levels after (ultra) marathon running (McCullough et al. 2011; Lippi et al. 2012; Mansour et al. 2017). However, the postexercise uncorrected uNGAL level after prolonged exercise found in our study (16.5 ng/mL) was lower compared to previous studies with postexercise values ranging from 37.6 to 47.0 ng/mL (McCullough et al. 2011; Lippi et al. 2012; Mansour et al. 2017). This might be explained by our relatively young population (23 years vs. >38 years), and the shorter period of exercise in our study (137 min vs. >240 min). Moreover, the uncorrected uNGAL levels in this study were far below the cutoff value (104 ng/mL) that has been used to diagnose kidney injury in clinical settings (Nickolas et al. 2012). Therefore, moderate intensity exercise in young individuals results in subclinical kidney injury.

### Clinical relevance

Our results demonstrate that healthy young male adults are well able to maintain eGFR_cystatin C_ after acute exercise, whereas an average decline of 15.4 mL/min/1.73 m^2^ (13.2%) and a largest decline of 43.2 mL/min/1.73 m^2^ (35.3%) was found after prolonged exercise. Our results suggest that prolonged exercise with ~3% hypohydration induces kidney stress, which result in kidney injury, as shown by increased osmolality corrected uKIM1 and uNGAL levels. Next to the detrimental effects of prolonged exercise and dehydration on kidney injury, previous studies also demonstrate that kidney injury might be influenced by heat stress, systemic inflammation, and renal perfusion(Smith et al. 1952; Junglee et al. 2013; Schlader et al. 2017). One might argue that the impact of heat stress, inflammation, and renal perfusion is higher after prolonged compared to acute exercise, and could influence our results. However, within this study we did not measure heat stress (core body temperature), systemic inflammation, and renal perfusion. Therefore, it is hard to establish whether the increase in kidney injury biomarkers can be explained by prolonged exercise with ~3% hypohydration solely, or by a combination of exercise duration, hypohydration, heat stress, inflammatory state, and renal perfusion. Future studies should therefore further elaborate on the relationship between exercise and kidney injury, in which more attention should be given to individual factors that influences kidney responses to exercise.

### Limitations

The strength of this study is the well‐controlled study design, in which subjects performed a continuous exercise bout at a constant workload with increasing levels of dehydration. However, there are some limitations that should be taken into account. First, by the absence of a measurement 24 h postexercise we were not able to determine whether the decline in kidney function and the induced kidney injury are temporary. However, previous studies demonstrated that the decline in kidney function and the increases in kidney injury markers already restored after 24 h of recovery (Lippi et al. 2008; McCullough et al. 2011). Second, spot urines were used for all laboratory analyses. Although spot urines correlate well with 24‐h urine samples and have the potential to operate as a surrogate for the preferred 24‐h urine collection, the use of spot urines is less accurate compared to a 24‐h urine collection (van Huysduynen et al. 2014). Moreover, the total urine volumes at baseline and after acute and prolonged exercise were not determined. Therefore, the urinary flow rate and urinary filtration over a period of time, which are potentially the best options to correct for changes in hydration status, cannot be calculated. Furthermore, within this study we did not measure core body temperature or inflammatory markers, while these factors can exacerbate kidney stress and elevate biomarkers for kidney injury.

In conclusion, our results suggest that, in a group of healthy young male participants, acute exercise barely impact on eGFR_cystatin C_ and biomarkers for kidney injury, whereas prolonged exercise is associated with a decline in eGFR_cystatin C_ and a further increase in biomarkers for kidney injury. Follow‐up studies are warranted to determine whether prolonged exercise‐induced acute kidney injury is primarily due to exercise duration, hypohydration, heat stress and/or inflammation.

## Conflicts of Interest

None.
